# In Vitro Ion Release of Wires in Removable Orthodontic Appliances

**DOI:** 10.3390/ma14123402

**Published:** 2021-06-19

**Authors:** Lena Wepner, Harald Andreas Färber, Andreas Jaensch, Anna Weber, Florian Heuser, Ludger Keilig, Lamia Singer, Christoph Peter Bourauel

**Affiliations:** 1 Oral Technology, Dental School, Medical Faculty, University of Bonn, 53111 Bonn, Germany; lena.wepner@t-online.de (L.W.); anna.weber@uni-bonn.de (A.W.); ludger.keilig@uni-bonn.de (L.K.); lamia.singer@yahoo.com (L.S.); 2 Institute for Hygiene and Public Health, Medical Faculty, University of Bonn, 53127 Bonn, Germany; Harald.Faerber@ukbonn.de (H.A.F.); Andreas.Jaensch@ukbonn.de (A.J.); 3 Department of Prosthetic Dentistry, Preclinical Education and Materials Science, Dental School, Medical Faculty, University of Bonn, 53111 Bonn, Germany; Florian.Heuser@ukbonn.de

**Keywords:** removable appliance, orthodontic wire, corrosion, cytotoxicity

## Abstract

Various orthodontic wire compositions and configurations are present on the market for removable appliances; however, there have still been only few studies focusing on the effect of resin color and additives such as glitter on corrosion of metallic wires under different conditions. Thus, the aim of the study was to compare concentrations of released ions (aluminium, chromium, nickel) in a corrosive medium under three different conditions: non-loaded wires, loaded wires, and non-loaded wires treated with Kukis^®^ cleaning tablets. Six different wires made of three types of steel alloy were embedded in PMMA resin leaving one centimetre of each wire emerging from the resin to come into contact with the corrosive medium. Glitter particles were added to half of the produced test specimens. For the unloaded test series, five specimens of each group were covered in a petri dish with 50 mL of corrosive medium (pH 2.3) following EN-ISO 10271 for seven days at 37 °C. The wires for the mechanically loaded test specimens overlapped the resin by 5 cm and were clamped into a time-switched electric drive for a defined period of time before the samples were taken after a testing time of 7 days. In the third group, unloaded test specimens were transferred from their petri dishes into the prepared Kukis^®^ solution every 24 h before being stored in the corrosive medium. Inductively coupled plasma mass spectrometry (ICP-MS) was used to quantify the specific ions in the corrosive solution. Statistical analysis showed that the mechanical loading of all wires could significantly raise the diffusion of ions into the corrosive medium. The colour of the resin did not affect the concentration of the released ions. The Kukis^®^ cleaning tabs could not lower the corrosion of the tested metals, as some of the wires were corroded even more using the brace cleanser. Glitter-containing test specimens showed significantly higher amounts of aluminium. Mechanical loading as well as the presence of glitter particles in the resin significantly affected ion concentrations.

## 1. Introduction

Removable orthodontic appliances are often used in orofacial orthopaedics to treat several kinds of jaw deformities and malocclusions. After the treatment is finished, fixed or removable retainers can be used to preserve the results of the therapy. The majority of patients are children and teenagers aged from six to sixteen years. The daily wearing time of the appliance (up to 23 h a day) can massively influence treatment speed and outcome. To maximize the patient’s compliance, orthodontists can offer various colours of resin and other additives, e.g., glitter particles embedded in the resin.

Metal ions such as chromium (Cr) and nickel (Ni) are released into the oral cavity due to corrosion of the wires and brackets of fixed orthodontic appliances under numerous environmental conditions [1]. Additionally, several mouthwashes can influence the corrosion of metallic appliances and cause the release of Ni and Cr into patient’s saliva [2].

Metallic appliances used in the oral cavity are repetitively challenged by acidic food and fluids (soft drinks, coffee) which usually initiate corrosion reaction and metal dissolution as well. Several studies concluded that the amount of released metal ions, corrosion potential and corrosion rates increased significantly when more acidic solutions (low pH values) were involved [3,4]. Lactic acid is considered one of the main metabolic substances existing naturally in the oral cavity, and is a by-product of oral tissues and bacteria. Thus, the role of lactic acid in the corrosion of metallic wires is important, as well, especially with respect to evaluating the effect of oral microbial-influenced corrosion (MIC) [5]. Moreover, some studies have stated that metallic wires show high resistance to corrosion in physiological saline and artificial saliva during immersion tests, while they corrode and discolour on exposure to lactic acid and formic acid [6,7].

Metals have been known to prompt potential allergic reactions for a long time, especially nickel. Grimaudo and Gölz et al. [8,9] showed that nickel has various effects on inflammatory processes and can even lead to gene expression in human monocytes, depending on its concentration. Bhasin et al. [10] showed that during the period of fixed orthodontic treatment, nickel and chromium levels elevated the gingival crevicular fluid (GCF) and showed increasing signs of gingival inflammation as well as oral mucosa cells [11]. Over the treatment period, serum levels can increase, although they do not reach toxic levels, as per the report of Quadras et al. [12]. Fitjer et al. [13] were able to prove that the wires inside of polymerised acrylic resin of removable appliances corrode, leading to discolorations of the resin and loss of sheen on the surface of the metal. Besides the above-mentioned health risks of common metals in orthodontic wires, aluminium (Al), as found in the glitter particles, is often criticized for causing DNA damage in fibroblasts [14], as revealed in in vitro studies. Aluminium is also under suspicion for causing neurodegenerative diseases, e.g., Alzheimer [15]. Although the concentration of dissolved ions from fixed appliances seems to decrease either constantly or even stops due to saturation [16], the release of critical elements in removable appliances should also be investigated. Thus, the aim of this study was to detect metal ions (aluminium, chromium, nickel) released from the wires into a corrosive solution during simulated daily usage.

## 2. Materials and Methods

In this study, three different types of alloys were chosen, each represented by two wires from two different manufacturers. As shown in Table 1, the first two wires were stainless chromium nickel steel alloys (Remanium^®^, Dentaurum, Ispringen, Germany; SS-Wire, American Orthodontics, Sheboygan, WI, USA), the third and fourth wires were made of manganese steel (Noninium^®^, Dentaurum, Ispringen, Germany; Menzanium^®^, Scheu Dental, Iserlohn, Germany) and the fifth and sixth wires were cobalt chromium nickel alloys (Elgiloy^®^, Rocky Mountain Orthodontics^®^, Denver, CO, USA; Remaloy, Dentaurum^®^, Ispringen, Germany).

Pieces of every type of wire were embedded in an orthodontic resin (Orthocryl^®^, Dentaurum, Ispringen, Germany) with different colours and combinations: colourless, colourless glitter (Orthocryl^®^ Disco-Glimmer, Dentaurum, Ispringen, Germany), blue and blue glitter. Under the different testing conditions (loaded, unloaded, Kukis^®^ cleaning tablets), these combinations were used in the current study to evaluate not only the effect of colour (colourless or blue) on the released Al, Cr, Ni ion concentrations, but also the effect of the presence or absence of glitter on the released ion concentrations. Silicone templates with the dimensions of 10 × 2.75 × 20 mm^3^ were used to achieve equal sizes for all test specimens. The total surface area of each test specimen was 5.65 mm^2^. EN-ISO 10271 for metallic materials was applied to prepare the corrosive medium at a pH of 2.3. The required ingredients were lactic acid (PanReac, AppliChem, Darmstadt, Germany), sodium chloride (Emsure^®^, Merck, Darmstadt, Germany) and deionised water (Aqua B. Braun, Ecotainer^®^, Melsungen, Germany). Immediately prior to use, the pH of the prepared solution was tested using a pH probe (pH 340i/set, WTW, Weilheim. Germany).

For the first test series (unloaded), a group of five test specimens was placed in petri dishes (Duroplan^®^, Schott, Mainz, Germany), then covered with 50 mL of the freshly prepared corrosive medium and subsequently stored in a climate test chamber (VEM 03/400, Heraeus Vötsch, Hanau, Germany) at an ambient temperature of 37 °C for seven days. A sample of 20 mL of each freshly prepared corrosive solution was kept as reference for future ion analysis. To prevent evaporation over the immersion time, the petri dishes were covered with several layers of Parafilm^®^ (Pechiney Plastic Packaging Inc., Chicago, IL, USA). After seven days, two samples of 20 mL of the corrosive solution were taken from each petri dish, transferred into welted glasses (Carl Roth^®^, Karlsruhe, Germany) and covered with a plastic lid.

The second test series remained mechanically unloaded, but Kukis^®^ cleaning tablets (ingredients see Table 2) were used. The prepared test specimens were again stored in a sealed petri dish with 50 mL of the corrosive medium for seven days at a temperature of 37 °C. The manufacturer’s instructions for Kukis^®^ tablet preparation were strictly followed in order to evaluate whether or not the cleaning tablets could lower the corrosion rate. After every 24 h, the five specimens of each group were taken out from the petri dishes with cleaned plastic tweezers. Specimens were carefully air dried to avoid loss of the corrosive medium. One Kukis^®^ tablet for every petri dish was dissolved in 200 mL of warm tap water (40 °C) in a single use plastic cup. The dried test specimens were placed in the prepared cleaning solution for 180 s before they were transferred into a plastic sieve.

In the sieve, the test specimens were cleaned of attached cleaning particles from the tablet for 40 s with running tap water at a temperature of 20 °C. The cleaned test specimens were dried with cotton paper and were returned to their respective petri dishes in each group. New layers of Parafilm^®^ were used after every cleaning procedure to avoid evaporation. After seven days, two samples (20 mL) of each petri dish were pipetted into glass containers and covered with a plastic lid.

**Figure 1:**
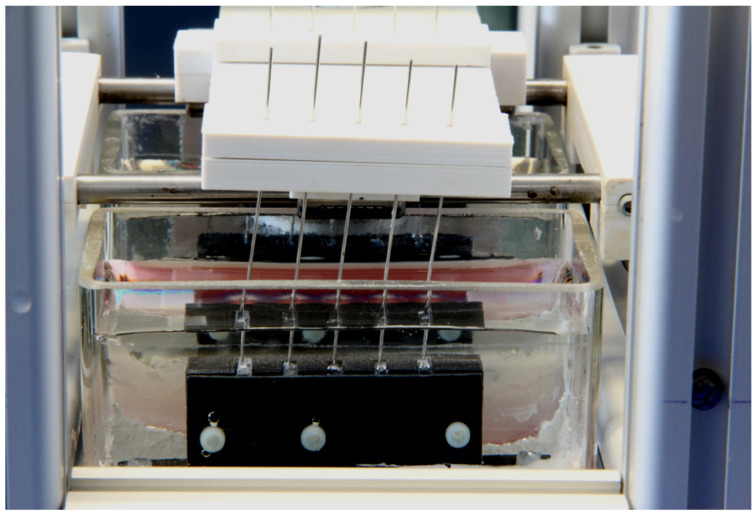
The photo shows five fixed test specimens during the loading process with a deflection of 3.5 mm.

For the third test series (loaded), a customised loading device was designed and manufactured using an in-house 3D printer (Renkforce RF100, Conrad, Hirschau, Germany). Inert polylactic acid polymer was used to print specimen holders and loading plates. Specimens were clamped in individual sample holders, which were placed in two cuboid glass containers (Duran^®^, Schott, Mainz, Germany). The wire extensions were hooked in a loading plate that could displace the wire ends in circular movements with a radius of 3.5 mm (Figure 1).

Test procedure: Five test specimens of each group were clamped in slots with the dimensions of the resin plates and tightened to avoid proper motion of the test specimens during the loading process (see Figure 1). The emerging ends of the wires were fixed in the electrically driven loading plate, controlled by a timer. The glass containers were filled with the prepared corrosive solution (125 mL) until the emerging ends were immersed for 1 cm. To minimise evaporation, the glass containers were covered with Parafilm^®^. The circular mechanical loading of the wires was performed as follows: one hour of loading at a frequency of 0.166 Hz, followed by a break of five hours, followed by the next loading cycle of one hour. This repeated loading was performed in the climate test chamber at 37 °C. The level of the corrosive solution was checked every 24 h and refilled with fresh solution when needed to maintain the level of 1 cm. The added volumes were recorded and used for calculation of released ions and statistical analysis. After seven days two samples of 20 mL from each glass container were taken for subsequent ion analysis.

An inductively coupled plasma mass spectrometer (ICP-MS 7700 Series, Agilent Technologies, Santa Clara, CA, USA) was used to analyse all collected samples for the following ions: aluminium, chromium, and nickel. The mass spectrometer measures the ion concentration three times per sample and delivers a mean value which is used to calculate the mean ion concentration of a test group. For example, one group consisted of samples from the corrosive solution of all uncoloured specimens that were compared to all coloured specimens. For the chromium and nickel concentrations, the number of compared samples was N = 14 for each group in terms of comparing the colour (blue vs. colourless), N = 8 for the comparison of the cleaning effect (Kukis^®^ vs. non Kukis^®^) and for the comparison of static vs. mechanic there were N = 16 and N = 12 samples, respectively. The number of the samples for the ion concentration of aluminium was different because each group was subdivided into glitter-containing and glitter-free samples. Consequently, the number N for the comparison of the colour was N = 7 (blue vs. colourless), N = 8 and N = 6 for (static vs. mechanic) and N = 4 for comparing the influence of the Kukis^®^ cleaning tablets.

The Kolmogorow-Smirnow test showed that the ion concentrations of the analysed samples were not normally distributed. Thus, a Kruskal-Wallis test with Bonferroni correction was subsequently applied for statistical analysis as a non-parametric test. With a global α = 0.05, statistical significance was revealed. The amounts of released aluminium, nickel and chromium ions were compared to each other in the three mentioned test series with respect to the following questions:

1. Does the colour of the orthodontic resin influence the ion release?
2. Does the presence of glitter particles influence the ion release?
3. Does the use of cleaning tablets lower the ion release?
4. Does the mechanical loading influence the ion release?

Moreover, the ion concentration of aluminium in the samples of those test specimens that contained glitter particles was compared to those that did not contain any glitter in the resin, in order to find out whether the aluminium of the glitter was leached out as well into the corrosive medium.

## 3. Results

### 3.1. Aluminium

**Figure 2:**
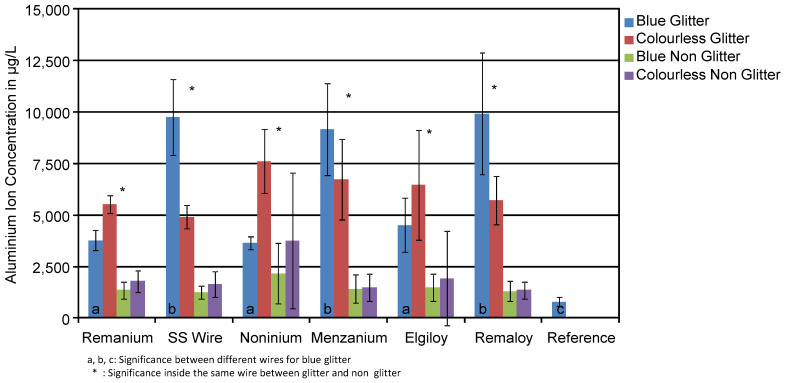
Mean aluminium ion concentrations in μg/L of the corrosion solutions of the different test specimens. The highest aluminium release for the glitter-containing specimens is obvious. The influence of the resin colour is minimal.

Figure 2 shows the mean concentration in μg/L of aluminium ions with their standard deviation found in the corrosion medium of transparent and blue test specimens with and without glitter, regardless of whether they were loaded or not. The statistical analysis showed that there was no significant difference of Al release in reference to the colour of the test specimens. Generally, Al concentration of the glitter-free groups was in the range of the reference solution (770 µg/L), while Al concentration of the glitter-containing samples was clearly increased. Remanium^®^ wires in combination with blue resin and glitter released 3764 µg/L on average, while colourless ones with glitter released 5511 µg/L. Conversely, Menzanium^®^ released more aluminium with the colourless resin (6270 µg/L), while the blue ones released 9164 µg/L.

Table 3 gives an overview of the measured Al mean ion concentrations in μg/L with their standard deviation found in the corrosive media with and without glitter particles. The glitter particles made of aluminium had a significant influence on the ion concentration with *p* < 0.0001 in the groups of SS wires, Menzanium^®^ and Remaloy^®^. On average, glitter-containing test specimens released 6467 µg/L aluminium, while the glitter-free ones released only 1260 µg/L over the period of a week.

**Figure 3:**
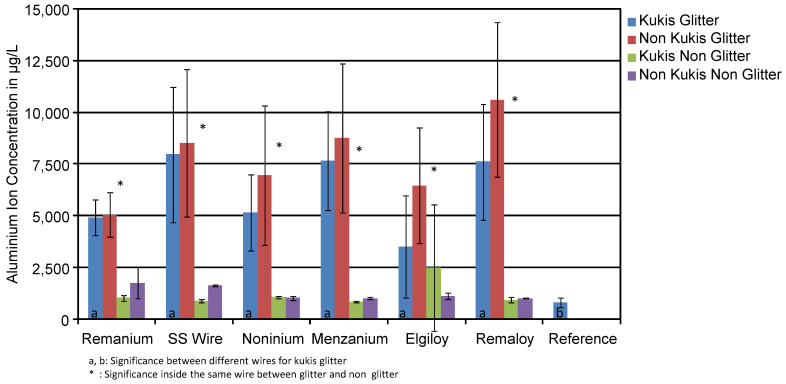
Mean aluminium ion concentrations in μg/L of the corrosion solutions of cleaned and non-cleaned test specimens. The cleaning procedure did not significantly influence the aluminium release.

The mean concentration of Al ions (μg/L) in the corrosive media of the cleaned daily (Kukis^®^) and non-cleaned test groups is shown in Figure 3. Glitter and glitter-free groups consisted of all samples with different colours, but mechanically loaded groups were discarded. There was no statistically significant difference in aluminium release between cleaned and non-cleaned specimens. Aluminium concentration in the corrosive medium of the glitter-containing test groups was clearly higher than in the glitter-free groups, as shown in Table 3.

The Elgiloy^®^ released 3507 μg/L for glitter-containing specimens cleaned with Kukis^®^ every day. In comparison, the Elgiloy^®^ wires without daily cleaning routine released 6451 μg/L. In this case, we can prove the reduction of aluminium release from the appliance. In general, test specimens cleaned with Kukis^®^ released overall 1260 μg/L, while the uncleaned ones released 1206 μg/L. There is only a slight difference in aluminium release from the orthodontic resin with the use of Kukis^®^.

**Figure 4:**
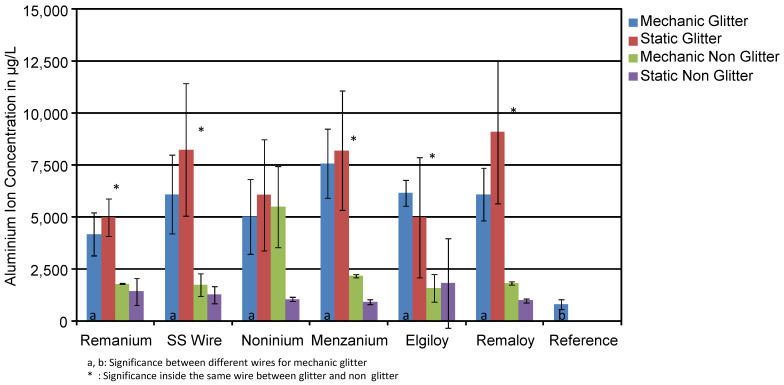
Mean aluminium ion concentrations in μg/L of the corrosion solutions of mechanically loaded and unloaded specimens. The effect of loading on the aluminium release was minor.

A comparison of mean concentrations (in μg/L) of Al ions found in mechanically loaded and unloaded test specimens is shown in Figure 4. Test groups consisted of blue and colourless specimens with and without glitter particles in the resin. Mechanically loaded specimens did release slightly higher amounts of aluminium than unloaded specimens in several selected groups. Typically, the effect of mechanical loading on the Al ion concentration was not significant. The loaded test specimen with the Elgiloy^®^ wire and glitter particles released 6158 μg/L, while the unloaded specimen released 4979 μg/L.

The glitter-containing test specimen with Menzanium^®^ released 7573 μg/L in the loaded test series and 8204 μg/L in the unloaded test series. This fact shows that mechanically loading of the wires does not show an effect on the migration of aluminium from the resin into the corrosive solution. Similar results can be found for the test specimen with Remanium^®^ wires. The loaded ones with glitter released 4176 μg/L in opposing to the unloaded ones with 4983 μg/L.

### 3.2. Chromium

**Figure 5:**
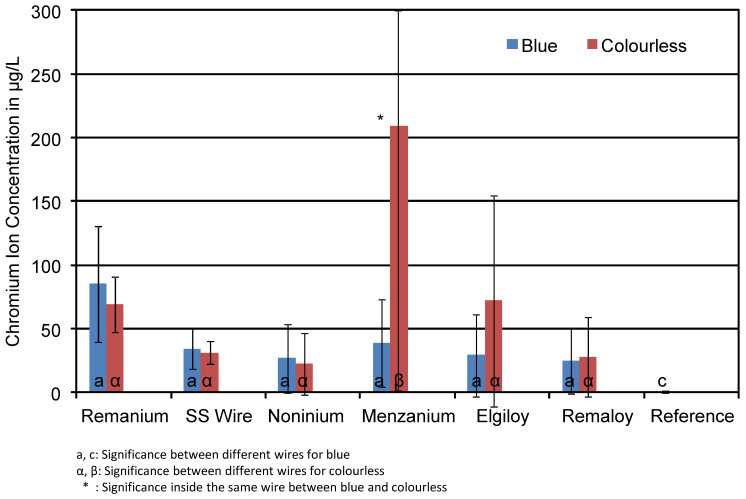
Mean chromium ion concentrations in μg/L in the corrosion solutions of the different test specimens. The influence of the resin colour on Cr concentration was not significant.

As the presence of glitter particles in the resin did not significantly influence the concentration of chromium ions in the corrosive solution (see Table 4), Figure 5 summarises the mean concentration of Cr ions found in the test solutions of the blue and transparent specimens without taking glitter content into account. The groups presented in Figure 5 consist of mechanically loaded and unloaded glitter-containing and glitter-free specimens. The colour does not significantly affect the corrosion rate of the wires in terms of Cr ions in the solutions. Compared to the reference (0.7 µg/L), all test groups showed increased Cr ion concentrations. A comparison of glitter-containing specimens and glitter-free ones can be taken from Table 4. The aluminium particles did not affect the chromium release. The presence of aluminium in the resin does not influence the release of chromium from the wire itself. SS wire released 19 μg/L chromium from the glitter-containing specimens and only 22 μg/L from the glitter-free ones. The colour (blue or colourless) did not influence the chromium release as well (43 μg/L vs. 31 μg/L). Menzanium^®^ showed very high chromium concentrations for around 208 μg/L in the colourless test group. This high mean concentration is evoked by the mechanically loaded colourless test specimens. Further evaluation is needed to check whether this can be considered as a mismeasurement of the ICP-MS or not.

**Figure 6:**
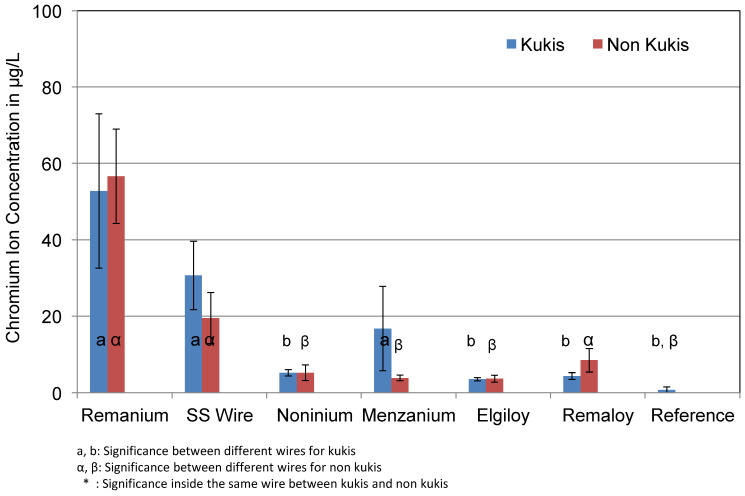
Mean Cr ion concentrations in μg/L of the corrosion solutions of cleaned and non-cleaned test specimens. The cleaning procedure did not significantly influence the chromium release.

Figure 6 shows the difference between specimens that were cleaned with Kukis^®^ tablets every 24 h and the specimens that were not cleaned during the test series. The effect of mechanical loading was not taken into account, and thus only corrosion solutions of unloaded specimens were compared. Test groups consisted of blue and colourless specimens. Similar to Al ion concentrations, the cleaning tablets did not show a significant effect on the corrosion rate or Cr ion concentration. The wire Remanium^®^ released 57.0 μg/L without daily cleaning, while the use of Kukis^®^ slightly reduced the concentration to 52.0 μg/L. Elgiloy^®^ and Remaloy^®^ both showed the lowest measured concentrations in the cleaned daily group (3.5 μg/L and 4.3 μg/L). Noninium^®^ released 5.0 μg/L chromium ions in the cleaned and non-cleaned groups. It needs to be discussed whether this slight reduction is of clinical relevance.

**Figure 7:**
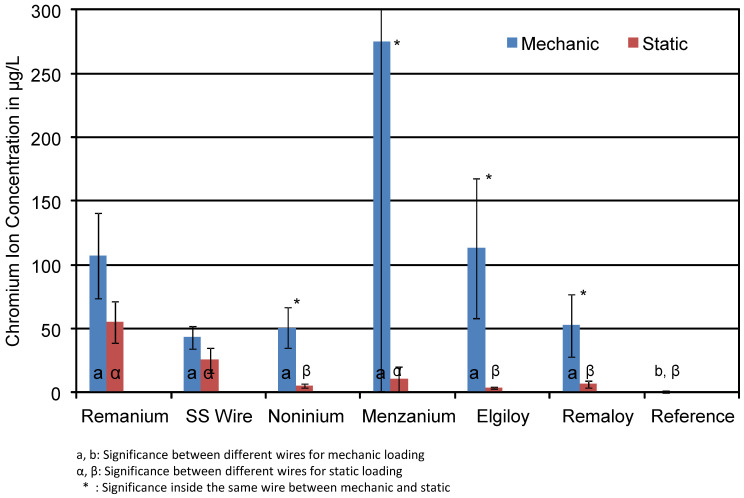
Mean Cr ion concentrations in μg/L of the corrosion solutions of mechanically loaded and unloaded specimens. The effect of loading on the chromium release is obvious. The * indicates statistical significance.

The mean concentrations of released Cr ions of the loaded and unloaded specimens are shown in Figure 7. Test groups were made up of blue and colourless specimens with and without glitter particles in the resin. Obviously, mechanical loading had an effect on the Cr ion concentration in the test solutions. The effect of mechanical loading for Remanium^®^ and SS Wires resulted in roughly doubled Cr ion concentrations (Remanium^®^: 54 and 107 µg/L unloaded versus loaded; SS: 25 and 43 µg/L). Noninium^®^, Remaloy^®^, Menzanium^®^, and Elgiloy^®^ showed significantly higher amounts of chromium in the loaded groups. All detected chromium concentrations were significantly higher than the Cr concentration in the reference solution.

### 3.3. Nickel

**Figure 8:**
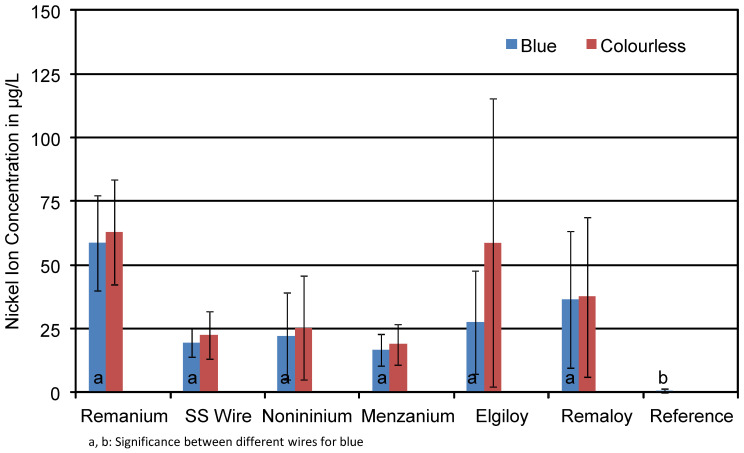
Mean nickel ion concentrations in μg/L of the corrosion solutions of the different test specimens. The influence of the resin colour is minimal.

As for the Ni concentrations, the presence of glitter particles in the resin did not significantly influence the concentration of nickel ions in the solution (see Table 5). Thus, in Figure 8, the mean concentration of Ni ions found in the test solutions is displayed for the blue and transparent specimens without taking glitter content into account. The groups presented in Figure 8 again consist of mechanically loaded and unloaded glitter-containing and glitter-free specimens. The colour did not significantly affect the corrosion rate of the wires in terms of Ni ions in the solutions. Compared to the reference (0.8 µg/L), all test groups showed clearly increased Ni ion concentrations. A comparison of glitter-containing specimens and glitter-free ones can be taken from Table 5. The aluminium particles did not significantly affect the nickel ion release. The CoCrNi wire Elgiloy^®^ showed concentrations around 48 μg/L for the glitter-containing specimens and 52 μg/L for the glitter-free ones. Minor differences can be found for the Remaloy^®^ wire as well: glitter-containing test specimens released 24 μg/L and glitter-free ones reported 28 μg/L. SS wire, as a stainless-steel alloy, released 19 μg/L for blue and 22 μg/L for colourless test specimens. It needs to be emphasised that the “nickel-free manganese steels” Noninium^®^ and Menzanium^®^ both released nickel ions into the test solution. Concentrations were around 25 μg/L for Noninium^®^ and 18 μg/L for Menzanium^®^ over the test period of a week.

**Figure 9:**
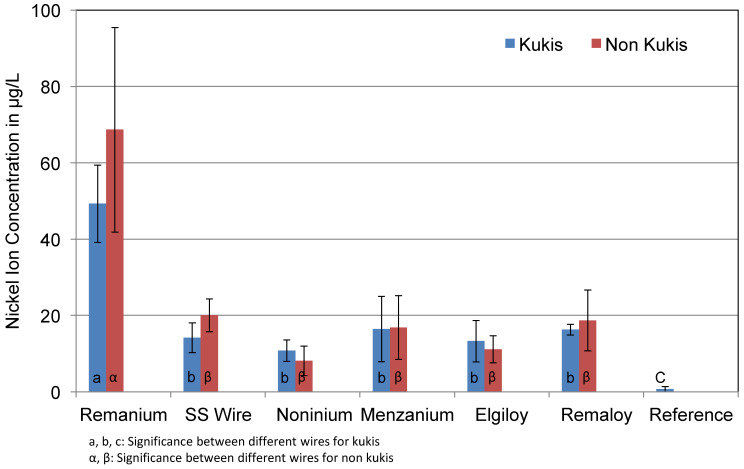
Mean Ni ion concentrations in μg/L of the corrosive solutions of cleaned and non-cleaned test specimens. The cleaning procedure did not significantly influence the nickel release. All Ni ion concentrations are clearly higher than the concentration in the reference solution.

Figure 9 shows the differences between the specimens that were cleaned daily with Kukis^®^ tablets and the specimens that were not cleaned during the test series. The effect of mechanical loading was not taken into account, and thus only corrosive solutions of unloaded specimens were compared. Test groups consisted of blue and colourless specimens. Similar to Al and Cr ion concentrations, the cleaning tablets did not show a significant effect on the corrosion rate or Ni ion concentration. The cleaned test specimens of the Remanium^®^ group showed concentrations of 48 μg/L on average, while the uncleaned wires released more nickel ions (68 μg/L). Slight difference in terms of nickel concentration can be found for the SS wire. The cleaned daily test specimens released 14 μg/L, whereas the non-cleaned specimens released 20 μg/L. No difference can be found for the Menzanium^®^ group, which showed an average concentration of 16 μg/L of nickel for both test series.

**Figure 10:**
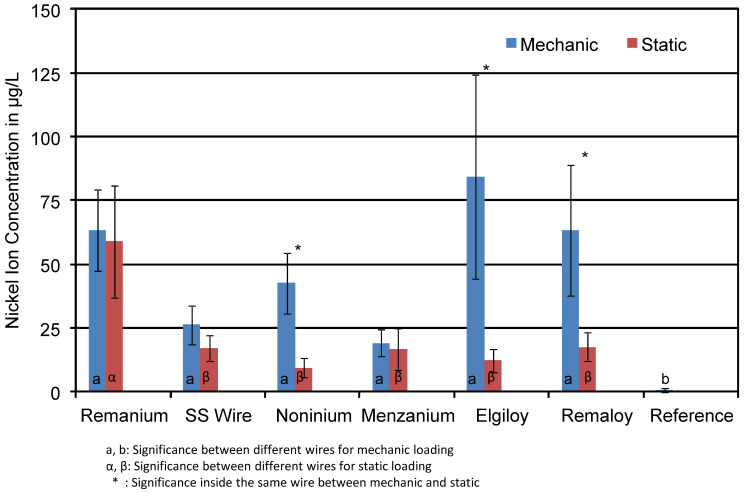
Mean Ni ion concentrations in μg/L of the corrosive solutions of mechanically loaded and unloaded specimens. The effect of loading on the nickel release is obvious. The * indicates statistical significance.

The effects of mechanical loading on the mean concentrations of released Ni ions are shown in Figure 10. Test groups consisted of blue and colourless specimens with and without glitter particles in the resin. A clear effect of mechanical loading was obvious only for four wires: SS wire, Noninium^®^, Elgiloy^®^, and Remaloy^®^. Mechanical loading resulted in an increase of the Ni ion concentration of 50% (SS), up to an increase by a factor of 7 (Elgiloy^®^). For Remanium^®^ and Menzanium^®^ wires, the effect of mechanical loading was minor. All detected nickel ion concentrations were significantly higher than the Ni concentration in the reference solution.

In the mechanically loaded group, the nickel concentration for Noninium^®^ wires was 42 μg/L, while the unloaded ones only showed concentrations around 10 μg/L. Another example for the high influence of mechanical loading can be seen in the Remaloy^®^ group. Loaded test specimens released 63 μg/L, while the unloaded specimens only showed concentrations of 17 μg/L.

## 4. Discussion

### 4.1. Materials and Methods

The tested wires and the orthodontic resin are common and often-used products for removable appliances. They fulfil the requirements of legislation for medical products. Nevertheless, the experiments revealed unknown facts about the material degradation and ion release of the simulated orthodontic appliance. The compatibility with in vitro conditions in the patient’s mouth must be critically discussed. The corrosive medium used here according to EN-ISO 10271 is significantly more acidic than the physiological pH of human saliva, which is around 6.5. However, the test conditions defined in EN-ISO 10271 for material testing of orthodontic appliances demand a pH of 2.3.

Ehrlich [17] has already shown that the use of Fusayama’s artificial saliva [18] in corrosion tests of fixed orthodontic appliances leads to lower but more realistic results. Thus, the direct transferability of ion concentrations at a pH of 2.3 to the patient is not necessarily a given. In addition to the total ion output, an evaluation of the daily output from the appliance is useful. According to Wendl, concentration decreases after the first few days of the appliance’s insertion and approaches saturation depending on the alloy [16]. The resin Orthocryl^®^ used in this experiment has already been classified as slightly toxic to fibroblasts in in vitro tests [19]. The decisive factor here was the amount of residual monomer in the appliance depending on the processing procedure. The additional use of aluminium-containing glitter particles in order to improve the children’s compliance and daily wearing time is an unnecessary additional exposure for this sensitive group of patients.

The simulated mechanical loading of the appliances in the current experiment aimed to represent the dynamic mandibular movements of a patient when speaking, opening the mouth, swallowing, inserting and removing the braces. The friction of the wires against the teeth, as well as the mechanical deflection of the wire retained in the acrylic resin, influence the ion release from the wires, as has already been demonstrated for fixed multiband appliances [20]. The cyclic loading of 60 min every 6 h in the experiment imitates the daily wearing time of such appliances. Due to the recommended wearing time of 18 h per day, a realistic loading duration was simulated.

Another point to be investigated was the reduction in corrosion claimed by the manufacturer on daily cleaning of the appliance with Kukis^®^. Such products are often used to remove adherent saliva components and tartar from the braces. Those unloaded test specimens that were cleaned daily with Kukis^®^ cleaning tablets should show whether the corrosion-reducing effect is true. However, the substances contained (citric acid, malic acid, peroxide) are known to promote corrosion processes [21,22]. Pitner showed, in a study in 1975, that the surface of the appliance is attacked and roughened, but there was no investigation of the influence on the corrosion of the wires [23]. In a further study [24], samples treated with Kukis^®^ even showed much higher ion concentrations than untreated samples stored in artificial saliva. A clinically relevant corrosion-reducing effect for the patient is therefore quite unlikely.

### 4.2. Aluminium

The results showed the concentrations of Al ions released into the solution under different test conditions. The high concentrations of Al ions found in the samples with glitter particles can be explained with the presence of aluminium particles in the resin of the respective test specimens. Although other test samples also contain smaller amounts of Al ions, there is a significant difference between test specimens with and without glitter for SS Wire, Menzanium^®^ and Remaloy^®^. The other wires also showed higher amounts of aluminium, but not on a significant level. Glitter-containing specimens reached concentrations of up to 14,474 μg/L, while solutions without glitter contained around 1,744 μg/L on average. This amount can be caused by traces of aluminium in the wires, although they are not mentioned in the available data sheets of the manufacturers. Furthermore, the loaded test specimens did not show a significantly higher amount of aluminium ions. This can be explained by the fact that the mechanical deflection of the wires in the loaded test series did not cause a relevant deformation of the glitter-containing resin that might have influenced the diffusion of aluminium ions from the resin into the corrosive medium. However, the wire deformation had an effect on the surface structure of the embedded wire and consequently altered its corrosion rate.

The used Kukis^®^ cleaning tablets could not lower the aluminium concentration significantly in any test series. The SS Wire and Remanium^®^ showed slight reduction of aluminium release with the use of Kukis^®^. The origin of the Al in the reference group (on average 770 μg/L) could be tracked for the water supply of the laboratory dish washer that was used to clean the petri dishes after each test series.

Especially because of the proximity of the released ions to the oral mucosa cells, these concentrations need to be evaluated critically [25]. The TWI (Tolerable Weekly Intake) recommended by the JECFA (Joint European Committee on Food Additives) for aluminium is 1.7 mg/kg/w and is strongly dependant on dietary habits [26]. For example, a bimaxillary appliance can have a surface up to 48 cm^2^ depending on the patient’s jaw size, this means an additional intake of 437 μg/L over the week. The authors want to emphasise that the use of the aluminium glitter particles for cosmetic reasons does not justify the unnecessary intake of ions through the treatment period which may last for several years.

### 4.3. Chromium

As Table 1 shows, all wires contain a high amount of chromium (11.5–22.0%). The mechanically loaded wires show significantly higher concentrations for Cr in the group of Elgiloy^®^. In general, the huge difference between the loaded and unloaded wires can be explained with alteration in the metal structure due to mechanical loading and deflection of the tested wires. As the diagram shows, large fluctuations can be seen in the results for loaded wires with regard to Cr. The Kukis^®^ cleaning tablets were not able to lower chromium levels in the non-loaded test series. The ingredients did not seem to have a remarkable influence on the diffusion of chromium ions into the corrosive solution. The pigments used in the resin of the blue test specimens with the Colour Index S.B 97 (C_36_H_38_N_2_O_2_) did not lead to higher or lower concentrations of chromium.

Similar to the known effects of aluminium on oral mucosa cells, Baričevic [27] showed that Co-Cr-Mo alloys used in removable prosthodontics can cause DNA damage in the mucosa cells as well [28]. Additionally, fixed orthodontic appliances containing chromium alloys lead to migration of these ions into mucosa cells of patients’ cheeks, as in vivo studies showed [29]. Other ions, such as titanium, showed less toxic effects on these cells compared to nickel and chromium, which were the least biocompatible ones. Although there is no tolerable weekly intake (TWI) for chrome stated by the EFSA in 2014 (European Food Safety Authority), awareness of long-term risks for children and teenagers is crucial.

### 4.4. Nickel

Similar to the other ions mentioned above, Ni is alloyed in each of the six wires. The significantly higher release for the wires Elgiloy^®^ and Remaloy^®^ in the loaded groups of test specimens can be deduced from the huge amount of Nickel in these wires (14–23%). Noninium^®^, as a manganese steel, also shows a significantly higher release of nickel in the loaded group. This has to be explained by the low percentage (<0.2%) of added nickel in the manufacturing process. Nickel is often alloyed into several steels to create acid resistance. After a short period of time, Ni builds up passive layers and lowers the corrosion process of ions in the solution. The mechanical stress is likely to damage this layer and makes it possible for ions to diffuse into the corrosive medium. The more Ni is added into the alloy, the more likely it will be to build up a stable oxide layer. As above, the Kukis^®^ cleaning tablets were not able to lower the Ni release in the different groups significantly. It seems that the ingredients of the cleaning tablets are either underdosed or some of the included substances may even boost the corrosion process, e.g., citric acid. The colour of the resin, either colourless or blue, did not affect the ion release. Moreover, the colour pigment of the used resin showed no influence on the detected metal ion concentrations. At this point, it needs to be mentioned that the two wires of manganese steel, Nonium^®^ and Menzanium^®^, both released nickel ions into the medium. Noninium^®^ released up to 61 μg/L in the mechanically loaded test series. This amount is way below the recent stated TWI of 13 μg/kg/w [30], but it shows that the manufacturer’s data do not completely fulfil the demands of a nickel-free product. The coating of wires either for removable or fixed appliances can reduce degradation of the wires [31], especially when we note that loaded wires release even more ions into the patient’s saliva. In mouse and rat models, high nickel intakes lead to genotoxic effects in animals.

In a longitudinal study [32] with patients wearing a fixed appliance for six months, the concentrations of nickel in gingival crevicular fluid (GCF) of some patients were high along with signs of gingival inflammation.

## 5. Conclusions

Obviously, mechanical stress greatly influences the release of ions into the corrosion test solution, not only for chromium, but also for nickel. Aluminium does not show higher concentrations in the mechanically loaded group because the glitter particles made of aluminium inside the resin do not get affected by the loading process. Nevertheless, we can see that there are high concentrations of aluminium in corrosive solutions of all test specimens that carry glitter particles. Concentrations reach up to 14,474 μg/L.

As Kuhta [33] already showed, the pH of the corrosion solution massively influences the amount of ions diffusing into the liquid. It needs to be checked whether the measured concentrations are transferrable to the conditions found in the oral cavity. We know that corrosion test series in artificial saliva show lower concentrations in comparison to the EN-ISO 10271 test solution [17]. Yet there is a correlation between apoptotic mucosa cells and the level of nickel as shown in in vivo studies [34,35]. Most likely, the diffusion of ions into the corrosion medium slows down after the first week of immersion, so we can assume that the ion levels drop under a critical value [36,37]. A perfect fit of the removable appliances will lower the forces on the wires and is likely to lower corrosion rates of the polymerised wires. We clearly need to evaluate whether it is necessary to provide removable orthodontic appliances with glitter particles to our patients considering that the wearing time of these appliances outlasts much longer than seven days. At this time, tests to evaluate biological hazards of these materials are voluntary and not mandatory. Moreover, it needs to be examined whether aluminium can migrate or influence oral mucosa cells at the pH that is found in oral cavity. The daily intake of aluminium over the mean time of treatment needs to be evaluated under these circumstances.

## Figures and Tables

**Table 1:** Alloy composition of the wires tested in this study (all compositions as given in manufacturers’ data sheets).

Manufacturer	Alloy	Composition in%									
		C	Si	Mn	Cr	Mo	Ni	P	S	Fe	Co
Dentaurum (Ispringen, Germany)	Remanium^®^	0.05–0.15	<2	<2	16–19	<0.8	6.0–9.5	<1	<1	rest	-
American Orthodontics (Sheboygen, WI, USA)	SS Wire	0.0–1.2	0–2	0–2	11.5–20	0.0–6.5	0–15	/	/	rest	-
Dentaurum (Ispringen, Germany)	Noninium^®^	<0.1	<1	16–20	16–20	1.8–2.5	<0.2	–	0.05	rest	-
Scheu Dental (Iserlohn)	Menzanium^®^	0.1	1	16–20	16–20	1.6–2.5	0.2	0.05	0.05	rest	-
Rocky Mountain Orthodontics^®^ (Denver, CO, USA)	Elgiloy^®^	0.15	–	1.5–2.5	19–21	7	14–16	–	–	rest	39–41
Dentaurum (Ispringen, Germany)	Remaloy^®^	0.03	<0.5	<0.1	18–22	3–5	19–23	–	<0.1	4–6	rest

**Table 2:** List of ingredients of the used Kukis^®^ cleaning tablets.

Kukis^®^ braces cleaner ingredients	Sodium Sulfate, Sodium Bicarbonate, Sodium Carbonate, Citric Acid, Malic Acid, Potassium Caroate, Sodium Carbonate Peroxide, PEG-150, Sulfamic Acid, Maltodextrin, TAED, PEG-90, Aroma, Sodium Chloride, Sodium Dodecylbenzensulfonate, Sodium Starch Ocetylsuccinate, Cellulose Gum, Methenamine, Cetylpyridinum Chloride, Aqua, CI 73015

**Table 3:** Mean concentration of aluminium ions in the unloaded test series of glitter-containing and glitter-free test specimens in comparison with the specific standard deviation in brackets. The * indicates statistical significance.

Wire Name	Analysed Element	Mean Concentration of Glitter-Containing Test Specimens in μg/L	Mean Concentration of Glitter-Free Test Specimens in μg/L
Remanium^®^	Aluminium	4638 (1007) *	1565 (504)
SS Wire		7322 (2835) *	1456 (514)
Noninium^®^		5613 (2322) *	2957 (2593)
Menzanium^®^		7934 (2376) *	1454 (646)
Elgiloy^®^		5484 (2250) *	1706 (1630)
Remaloy^®^		7814 (3067) *	129 (436)
Reference			770 (263)

**Table 4:** Mean concentration of chromium ions in the unloaded test series of glitter-containing and glitter-free test specimens in comparison with the specific standard deviation in brackets. No significant differences can be found.

Wire Name	Analysed Element	Mean Concentration of Glitter-Containing Test Specimens in μg/L	Mean Concentration of Glitter-Free Test Specimens in μg/L
Remanium^®^	Chromium	65.3 (12.8)	56.3 (24.0)
SS Wire		19.9 (3.6)	22.0 (10.2)
Noninium^®^		20.9 (13.9)	26.4 (22.56)
Menzanium^®^		18.4 (7.2)	16.9 (7.1)
Elgiloy^®^		40.4 (37.5)	45.8 (52.1)
Remaloy^®^		35.0 (27.6)	39.2 (30.4)
Reference			0.7 (0.7)

**Table 5:** Mean concentrations of nickel ions in the unloaded test series of glitter-containing and glitter-free test specimens in comparison with the specific standard deviation in brackets.

Wire Name	Analysed Element	Mean Concentration of Glitter-Containing Test Specimens in μg/L	Mean Concentration of Glitter-Free Test Specimens in μg/L
Remanium^®^	Nickel	85.7 (45.2)	68.5 (22.1)
SS Wire		36.1 (10.2)	29.5 (14.6)
Noninium^®^		20.3 (21.3)	29.0 (28.3)
Menzanium^®^		24.4 (22.8)	30.2 (38.6)
Elgiloy^®^		48.7 (58.2)	52.2 (74.0)
Remaloy^®^		24.5 (24.6)	27.7 (32.0)
Reference			0.8 (0.8)

## Data Availability

Data available on request due to restrictions e.g., privacy or ethical. The data presented in this study are available on request from the corresponding author. The data are not publicly available due to formal regulations regarding a doctoral thesis.
